# Prediction of Deleterious Single Amino Acid Polymorphisms with a Consensus Holdout Sampler

**DOI:** 10.2174/0113892029236347240308054538

**Published:** 2024-03-14

**Authors:** Óscar Álvarez-Machancoses, Eshel Faraggi, Enrique J. deAndrés-Galiana, Juan L. Fernández-Martínez, Andrzej Kloczkowski

**Affiliations:** 1 Group of Inverse Problems, Optimization and Machine Learning, Department of Mathematics, University of Oviedo, C. Federico García Lorca, 18, 33007, Oviedo, Spain;; 2 School of Science, Indiana University–Purdue University Indianapolis, IN, USA;; 3 Department of Computer Science, University of Oviedo, C. Federico García Lorca, 18, 33007, Oviedo, Spain;; 4 Institute for Genomic Medicine, Nationwide Children’s Hospital, Columbus, OH, USA;; 5 Department of Pediatrics, The Ohio State University, Columbus, OH, USA

**Keywords:** Polymorphisms, holdout sampler, protein mutation, deep sampling, machine learning, single amino acid variants

## Abstract

**Background:**

Single Amino Acid Polymorphisms (SAPs) or nonsynonymous Single Nucleotide Variants (nsSNVs) are the most common genetic variations. They result from missense mutations where a single base pair substitution changes the genetic code in such a way that the triplet of bases (codon) at a given position is coding a different amino acid. Since genetic mutations sometimes cause genetic diseases, it is important to comprehend and foresee which variations are harmful and which ones are neutral (not causing changes in the phenotype). This can be posed as a classification problem.

**Methods:**

Computational methods using machine intelligence are gradually replacing repetitive and exceedingly overpriced mutagenic tests. By and large, uneven quality, deficiencies, and irregularities of nsSNVs datasets debase the convenience of artificial intelligence-based methods. Subsequently, strong and more exact approaches are needed to address these problems. In the present work paper, we show a consensus classifier built on the holdout sampler, which appears strong and precise and outflanks all other popular methods.

**Results:**

We produced 100 holdouts to test the structures and diverse classification variables of diverse classifiers during the training phase. The finest performing holdouts were chosen to develop a consensus classifier and tested using a k-fold (1 ≤ k ≤5) cross-validation method. We also examined which protein properties have the biggest impact on the precise prediction of the effects of nsSNVs.

**Conclusion:**

Our Consensus Holdout Sampler outflanks other popular algorithms, and gives excellent results, highly accurate with low standard deviation. The advantage of our method emerges from using a tree of holdouts, where diverse LM/AI-based programs are sampled in diverse ways.

## 1 INTRODUCTION

Distinguishing residue substitutions that affect protein function and lead to medical disorders is one of the most important problems of present-day molecular medicine [1]. Most (98.5%) of the human genome comprises of noncoding DNA, and the majority of variants are located in the noncoding part of the genome. Furthermore, the majority of mutations are neutral and do not influence the phenotype. This constitutes evolutionary conservatism, which results from the fact that protein structure is much more conserved during evolution than protein sequence. In our paper, we will concentrate on single nucleotide variations (SNV) in protein-coding genes. There are two types of SNVs within protein-coding genes: synonymous SNVs (sSNVs), which is a consequence of the degeneracy of the genetic code since 20 amino acids are coded by 64 triplet codons, and nonsynonymous SNVs (nsSNVs). For sSNVs, a nucleotide mutation does not lead to residue change, while for nsSNVs, a nucleotide mutation results in residue change. It has been found that each individual human genome contains 24,000 - 40,000 residue variations [2] coming from nsSNVs [3, 4]. Whereas most residue mutations are impartial [5, 6], some mutations have an impact on protein function. These impacts can lead to mutation-related diseases [7]. In spite of the fact that most of the variations within the GWAS Catalog are in the noncoding genomes, generally, half of known disease-related variations in protein-coding genes are due to nonsynonymous variations [8, 9]. Subsequently, it is vital to predict the impact of the variation on the protein function and to distinguish between neutral and deleterious mutations [10]. It is well known that some mutations might be straightforwardly linked to medical disorders such as cancer, or Parkinson's and Alzheimer's disease [11]. Despite the huge amount of medical data collected and enormous efforts to reveal links between the genotype and the phenotype, the problem is still unsolved due to the complexity of the basic issue. Subsequently, computational ML/AI-based strategies have been developed to analyse nsSNVs information.

Fig. (**1**) presents a general scheme of the work: the goal is to combine sequence information and big data analytics to predict the consequence of protein mutations to distinguish between neutral and deleterious variations.

A wide variety of computational methods have been developed to predict the effect of residue mutations on protein function. Of particular interest are AI/ML-based and classification-based methods that combine different types of biological data to distinguish between harmful and neutral residue mutations [12]. The use of AI/ML methods is required because the relation between residue mutations and their effects on phenotype is *a priori* unknown.

Some of these methods use evolutionary information to distinguish whether a particular residue variation is harmful or neutral, such as Provean [13, 14], SIFT [15], PANTHER [16] or the Evolutionary Action method [17, 18]. The main disadvantage is that they use evolutionary conservatism information for a single position in multiple sequence alignments. Other methods combine physicochemical properties with evolutionary and sequence data to improve predictions. This includes Mutation Taster [19], CADD [20], Polyphen-2 [21], SNPandGO [22], PhD-SNP [23], PredictSNP [24] or MAPP [25]. These methods use a number of ML procedures to forecast the impact of missense mutations. Nevertheless, these methods often have a high rate of false positives, *i.e*., the predicted harmful variants are frequently in reality neutral [26]. Although the structure, sequence, and evolutionary information are important factors for protein function, it is rarely used in classification methods [27-29]. Lastly, another significant disadvantage of these supervised ML-based techniques is that decision rules result from overlying data in training sets, which leads to performance overestimation [30, 31]. Therefore, it is highly recommended to use completely independent sets of data to train, test, and validate all prediction techniques [32]. This issue is additionally exacerbated by the absence of variability in the training data sets, which leads to biased forecasts [33].

To evade these restrictions, here we tried to forecast the impact of mutations by increasing the sampling of protein properties to understand the relation between the stability and harmfulness of mutations. In addition, to avoid biases, the data are randomly separated into diverse holdouts, where training and testing are accomplished by random selection of features of nsSNV. We then select the settings that offer the best performance and combine them to create the consensus classifier. It is commonly known that different classifiers might be grouped to form a consensus classifier [34], such as Meta-SNP [35], CONDEL [36] or PredictSNP [24].

Our consensus classifier outperforms each separate classifier and provides a robust and comprehensive prediction system able to identify and analyse protein properties that better categorize residue variations according to their harmfulness.

## 2 MATERIALS AND METHODS

### 2.1 nsSNV Datasets

A set of neutral and deleterious mutations was derived from the UniProt/SwissVar database [37] to train, test and validate the prediction methodology. We used k-fold (1 ≤ k ≤5) cross-validation, in which the 5th fold was applied for blind validations, and the remaining data were used for training by creating 100 random holdouts, with 75% of them utilized for training and 25% for testing. The entire set of mutations contained 38,460 single-point mutations from 9,067 proteins. (See Supplementary Material **1**).

### 2.2 The Holdout- nsSNV Methodology

Prediction of the consequence of nsSNVs involves substantial uncertainty, and we need to correctly sample the uncertainty space while performing effective classification.

Generally, any classification procedure can be represented by a simple linear regression. The least-square fitting of a linear model involves finding a set of parameters 𝓶 = (𝑎_0_, 𝑎_1_), that the Euclidean distance between the vectors of observed data 
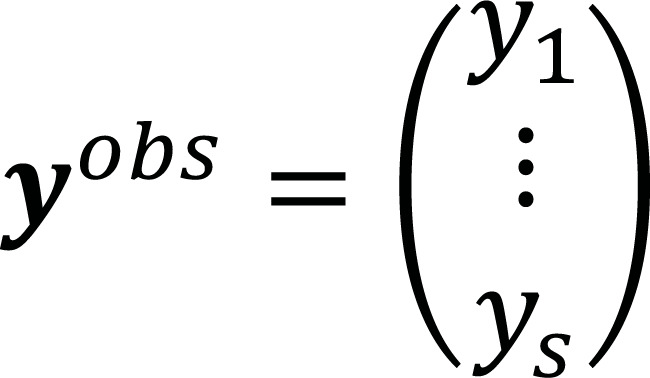
 and the predicted data 𝒚^*pre*^ (𝑚) = 
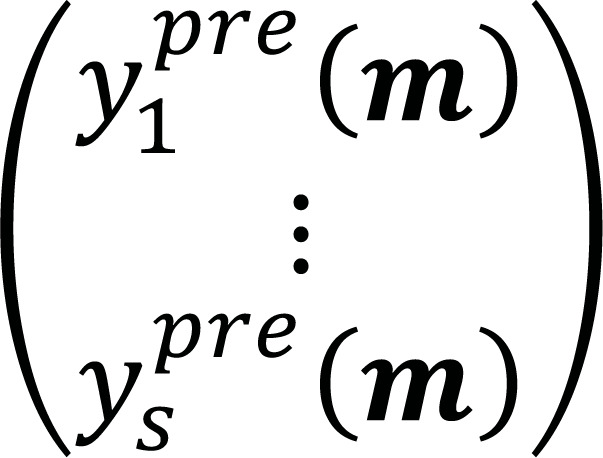
 is minimized, where S is the dimension of the space. In a matrix form, we need to solve a linear system of equations 𝐹𝓶 = 𝒚^*obs*^, where the matrix 𝐹 = [1_𝑹^𝒔^_, 𝒙] is built from the coordinates of the data points 
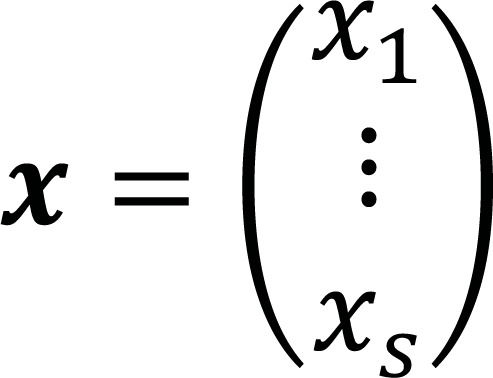


The analysis of the uncertainty is based on sampling the set of models 𝓶 = (𝑎_0_, 𝑎_1_), that forecast 𝒚^*obs*^ within an accepted tolerance error, 𝘔_*tol*_ (Eq. 1):


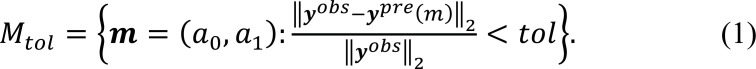


Fernández-Martínez *et al.* [38-40] have shown that in the case of linear problems, the cost function has the topography of a straight, flat valley. For the nonlinear cases, the topography of the cost function contains curvilinear valleys joined by saddle points [38-40].

Fig. (**2**) illustrates a simple 2d regression model by showing ellipses of uncertainty for different relative misfits and varying sets of parameters associated with different bagging experiments.

The problem of predicting the effects of nsSNVs can be viewed as a generalized regression problem between the sets of discriminatory nsSNV features describing the result of mutation and the set of classes in the training set of data. This analogy with the linear prediction problem (least squares) is very important to understand the algorithm used here.

This procedure has been successfully applied by us to predict protein stability changes upon single and multiple residue mutations [41], and phenotype prediction and for the sampling of defective pathways in Alzheimer’s disease [42], Multiple Myeloma [43], and Inclusion Body Myositis [44, 45]. The Holdout sampler terminology introduced here means the 5-fold cross-validation procedure that involves data pre-processing in holdouts.

Here, we introduce a prediction algorithm based on consensus methodology using holdouts [46] and combining an Extreme Learning Machine (ELM) with a Random Forest (RF) procedure [47].

A k-fold (1 ≤ k ≤5) blind cross-validation is performed by using 75% of the PDB data to create 100 holdouts. Each holdout constitutes a data bag, with 75% of the data applied for training and 25% for testing purposes. For each holdout a random selection of features and classification activation function was used to avoid overfitting or underfitting problems and to ensure the robustness of the methodology.

The Holdout- nsSNV algorithm predicts the effect of residue substitutions in proteins by categorising them as neutral or deleterious by combining sequential, structural, and evolutionary information. The algorithm avoids overfitting and underfitting problems.

The protein sequence profile is calculated by utilizing the Protein-Protein Basic Local Alignment Search Tool (BLASTP) algorithm [48] by searching for homologous protein sequences in the UniRef90 database in UniProt [37] with default parameters and a threshold e-value of 10^-9^. From each BLASTP run, we get (i) the sequence alignment score, (ii) the alignment ratio and (iii) the number of aligned sequences. The algorithm also calculates (iv) the sequence profile in the form of a 20-value array that shows the frequency of each of 20 naturally occurring amino acids at every position in the protein sequence. Lastly, (v) the mutation vector is calculated, defined as a vector of 20 values corresponding to each amino acid, where the wild-type residue is assigned the value -1, the mutated one a value of +1, and the non-mutated ones the value of 0 at the given position, respectively.

The evolutionary information is retrieved from the PANTHER (Proteins Annotation through Evolutionary Relationships) database, which evaluates the preservation time of a given residue at a given position in phylogenetic trees; longer preservation time indicates a larger functional impact [49, 50]. The structural information is the Solvent Accessible Area computed for a given protein structure with the ASAquick tool [51]. The Holdout-nsSNV procedure combines all this information in a matrix form and utilizes it for training, testing and validation.

### 2.3 Consensus Holdout Training and Selection

Usually, holdout-based methods are used for model validation (R, 1995). Nevertheless, here, we propose a different approach in which holdouts are additionally employed to optimally perform a consensus classification at the learning stage. This approach, based on the idea of bootstrapping (Efron B, 1993), has been earlier fruitfully used by us for phenotype prediction [52].

We construct a classifier 𝐿*, that links the nsSNVs attributes and the set of two classes {Deleterious, Neutral} dividing the mutants (Eq. 2):


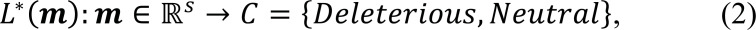


with *s* being the length of the attribute that has been selected for the classifier 𝐿*.

We have to find discriminatory attributes of the nsSNV corresponding to 𝐿*(𝓶) that better fit the observed class vector ***c^obs^***. Subsequently, modeling the consequence of nsSNVs consists of two steps: learning and validation. The learning stage comprises the selection of a subset of the training data set with a known class vector ***c^obs^***; *i.e*., finding the minimal subset of attributes that maximizes the learning accuracy (Eq. 3):


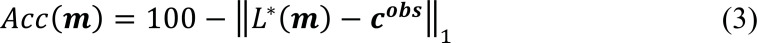


where ||𝐿*(𝓶) - ***c^obs^***||_1_ means the prediction error in the L_1_ robust norm.

According to Bayes's rule (Eq. 4):


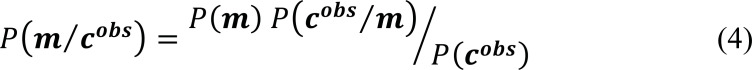


where 𝑃(𝓶) is named the prior probability, 𝑃(***c^obs^***/𝓶) denotes the likelihood, and 𝑃(***c^obs^***) means the evidence. The term 𝑃(***c^obs^***/𝓶) depends on the model accuracy *Acc*(𝓶).

This approach is based on the statistical method of bootstrapping, or arbitrary sampling with replacement [46], which is used to create confidence intervals and to estimate the sampling distribution of any statistics *via* a random sampler. In our previous works, we used this methodology to optimally sample model parameter posterior distribution by the least squares fitting of different data bags [53-55].

The Holdout algorithm is composed of three stages:

- Data bagging: We randomly divide the data into holdouts, where 75% of the data are used for learning and 25% for testing/validation. In this work, 1000 different bags were generated, in each bag, a random selection of attributes (𝓶_*i*_) is performed before the classification of the proteins *p_k_*, according to the observations, ***c^obs^***.
- Data Testing: After the completion of the learning stage, testing is performed to calculate the prediction accuracy of each holdout. Subsequently, we obtain a distribution of holdouts according to their predictive accuracy and select those performing best.
- Holdout Selection: Once all mutants are classified using the testing data set, the holdout accuracy is calculated, and the best holdout predictors are used for blind validation. The holdouts that fulfill the condition: *Acc_HD,i_* > 0.90. *Acc_HD,max_* are selected for the blind validation.

This algorithm is used with the ELM and Random Forest as classifiers.

#### 2.3.1 Extreme Learning Machines

The concept of Extreme Learning Machines ELM was proposed by Guang-Bin Huang in 2006 and became very popular in machine learning due to its very fast and efficient method of tuning the parameters of hidden nodes in a neural network.

ELM consists of a single or multiple layers of hidden nodes with hyper-parameters [56]. The weight of each hidden neuron can be learned [57]. ELMs can be trained faster than neural networks employing backpropagation by using the Moore-Penrose pseudoinverse [58]. Guang-Bin *et al.*, demonstrated that ELMs could outperform Support Vector Machines (SVM) models, since SVM could provide suboptimal solutions in regression and classification problems [59, 60].

The input weights for the ELM are selected randomly, and the output weights are computed by solving a system of linear equations. Furthermore, ELM could also be used to select a linear or non-linear activation function (sigmoid and sinusoidal) [61, 62].

Here, the architecture of ELM is undefined; subsequently, at each holdout, it is randomly sampled, trained, and tested. The ELM prediction is written as follows (Eq. 5):


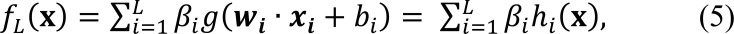


where *L* is the number of hidden neurons, and *h_i_*(**x**) stands for the output of the *i*-th hidden node that has the form *h_i_*(**x**) = *g*(*w_i_*. **x** + *b_i_*), with *g* being the activation function and *w_i_* a set of randomly chosen weights of the same size of **x**, to perform the inner product *w_i_*. **x**, and *b_i_* is the bias of the *i*-th neuron. In our algorithm, the activation function *g*, and the number of hidden layers *L* are assigned at each holdout randomly [56].

The methodology consists of giving a training dataset of size *m* whose classes are known and corresponding to different signatures **x**_*j*_, and observed classes *y* = [*y_1_*  *y_2_* ... *y_m_*]^*T*^, finding ***β*** = [*β_1_*  *β_2_* ... *β_L_*]^*T*^, such as (Eqs. 6 and 7):


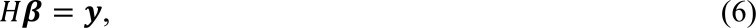


with


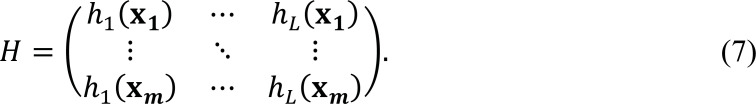


Linear system (6) is solved in the least-squares sense *via* the Moore-Penrose pseudoinverse [56].

#### 2.3.2 Random Forests

In addition to ELM, we used Random Forests (RF) in the consensus prediction. Random Forest is one of the most popular machine learning methods proposed in 1995 by Ho *et al*., utilizing an ensemble learning technique based on a multitude of decision trees for classification and regression [62].

The Random Forest uses a bootstrap method for each holdout; therefore, a data bagging procedure (Random decision tree) is performed for each data holdout (bag). For a given training set *T_x_* = {***x**_1_*, ***x**_2_*, ... , ***x**_s_*} and a vector of observations 
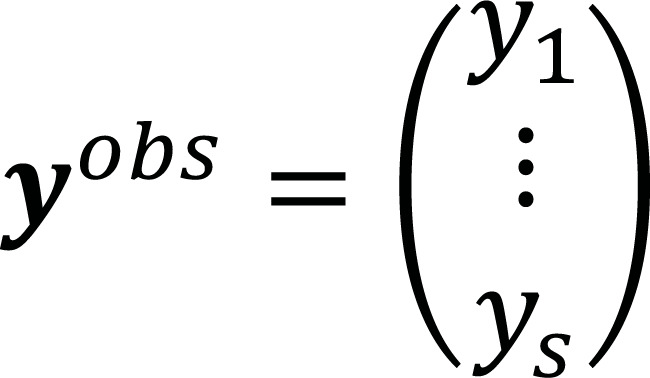
, which are bagged repeatedly, RF selects a completely random sample with replacement within the training data set in order to fit trees to this sample. Based on this, several classification trees are trained, and the overall prediction could be obtained by computing the average prediction of each B tree, 
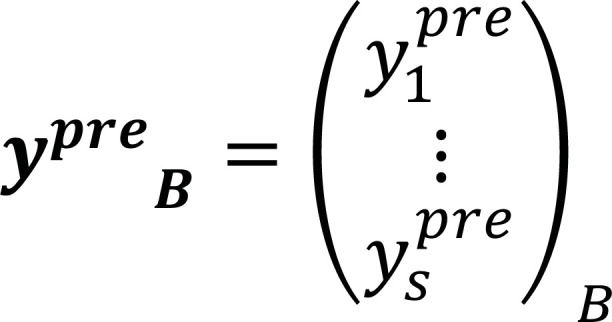


While the prediction for each tree is highly sensitive and noisy, and these individual predictions are diverse and uncorrelated, the global average over all the trees is robust and accurate with highly reduced bias.

## 3 RESULTS

The Holdout- nsSNV algorithm combines sequential, structural, and evolutionary features and stability attributes. The algorithm uses the sequence of a given mutant protein, determines all the attributes, and performs the classification by using the Holdout Sampler as a consensus classifier. Consensus classifiers rely on the principle of Condorcet; for independent decision-makers, the dominant decision tends to be right when the number of decision-makers increases. Consensus classifiers are accurate and reliable alternatives to individual and traditional ML-based algorithms [24].

Table **1** presents the performance of our Holdout-nsSNV algorithm:

We observe a high dispersion and uncertainty in the testing accuracy for individual holdouts, as seen in Fig. (**3**). The cumulative distribution function for the Holdout Sampler clearly shows the occurrence of two classes of holdouts in terms of accuracy.

When the holdouts with higher performance are selected, by applying a threshold *Acc_i_* > 0.90. *Acc_i,max_*, the algorithm is blindly validated using a *k*-fold (1 ≤ *k* ≤5) cross-validation procedure to ensure that most of the variations across the datasets are utilized both in the training and in the blind validation. Table **2** presents the results of blind validation for each individual K-Fold (Supplementary Materials **2**-**6** show additional data for each K-Fold):

As expected, the algorithm is very robust, providing similar accuracies regardless of the K-Fold. This is further supported by the fact that the median and the average accuracies are very close, combined with low standard deviations and low uncertainties (interquartile range). The cumulative distribution function of accuracies in blind validation is presented in Fig. (**4**), which shows smooth curves within a very small range of possible accuracies.

**Fig. (5):**
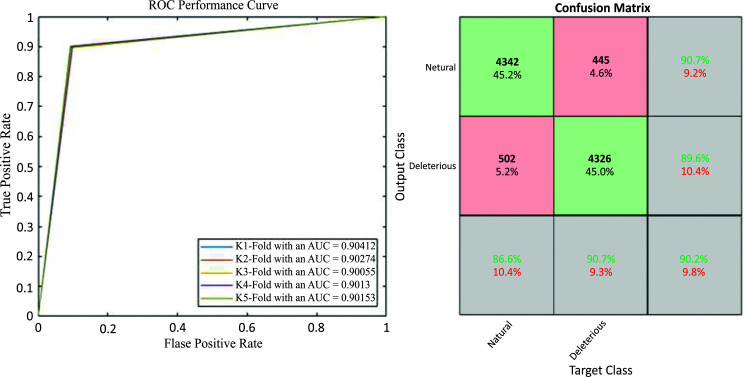
(Left). Holdout sampler overall receiver operating characteristics curve for each K-Fold. We also show the different groups of the confusion matrix. The number of examples in the False groups (false positive and false negative) are very balanced (445 *vs* 502).

**Fig. (6):**
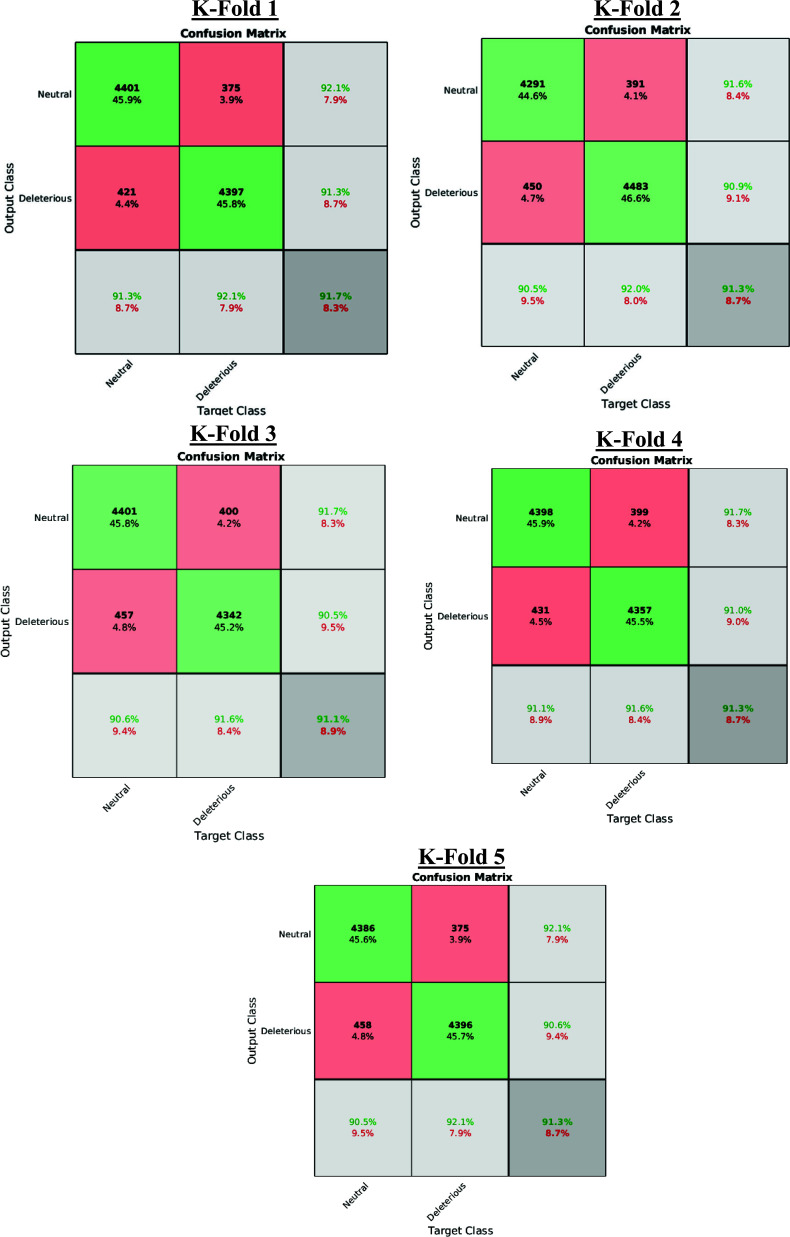
Confusion Matrix for each specific K-Fold. In each confusion matrix table, the rows indicate the predicted class *via* the Holdout Sampler, and the columns correspond to the targeted classes. The diagonal cells (green colour) refer to the observations that are correctly classified, whereas the red diagonal cells in red refer to those that are not properly classified with the Holdout Sampler. Both the number of observations and the percentage of the total number of observations are shown in each cell. The third column represents the percentage of all the examples predicted to belong to each class (properly predicted in green and wrongly predicted in red). This metric is sometimes referred to as precision and false discovery rate. The third row shows the percentages of all the examples belonging to each class that are correctly and incorrectly classified. These parameters are also known as recall and false negative rates. The cell in the bottom right shows the overall accuracy.

Additional information about the performance of the algorithm for cross-validated testing is shown in Figs. (**5** and **6**), which display a Receiver Operating Characteristic (ROC) performance curve (Fig. **5**) and the Confusion Matrix (Fig. **6**), respectively. In summary, the algorithm gives an overall accuracy of 90.2% and an average value of Mathews Correlation Coefficient (MCC) of 0.80 with the Areas Under the Curve (AUCs) that range between 0.9005 and 0.9041.

In addition to providing a robust method to predict the effects of nsSNVs, this manuscript aims to provide benchmarks to describe the performance of the algorithm and benchmarks for possible future development.

First, as pointed out in Section 2, the cumulative distribution function of accuracy of the Holdout Sampler experiences a sharp increase around the 50^th^ percentile. This feature is due to the difference in accuracy between the holdouts that use ELM and RF as classifiers. Fig. (**7**) shows the cumulative distribution functions of accuracies for training and testing for the holdouts separately employing ELM and RF classifiers.

It is possible to observe in each subplot that the dispersion of accuracies for the RF holdouts is much smaller than for the ELM holdouts. This result is expected since RF is a consensus classifier itself, which reduces bias while increasing robustness and accuracy. On the other hand, the accuracies for ELM holdouts are more dispersed because they are subjected to more bias when varying the decision boundaries (different on each holdout). This is especially critical for the calculation of the ELM Moore-Penrose pseudoinverse, which is sensitive to attributes that introduce noise in the classification learning process.

In addition to the classifier utilized, the selection of attributes or boundary conditions plays a crucial role in the overall performance of the algorithm. Deep learning algorithms perform very well when boundary conditions introduce limited variability or bias. To avoid this problem, we proposed an approach that can be considered as deep sampling. We perform a training stage of a deep learning algorithm without fixing the architecture of the classifier and by a random selection of the attributes utilized to infer the class. This feature is clearly observed in Fig. (**8**), which depicts the frequency (posterior analysis) of the high discriminatory attributes (Evolutionary, Mutation, Sequence Profile, Sequence, Stability, Structural attributes) identified in the sampling for the ELM or RF classifiers. Mutation and sequence attributes are the most frequently sampled discriminatory attributes regardless of the classifier. Conversely, the stability seems to be the less crucial (or sampled) in high performing holdouts. One of the advantages of the holdout sampler is that it allows this kind of posterior analysis.

Our deep sampling approach utilizing the Holdout Sampler helps us to intuitively discriminate variables that are the most discriminatory. Histogram plots in Fig. (**8**) show that for high-performing holdouts, we observe more frequent presence of certain attributes, such as mutation, sequence, and structural features, which are the most determinant, regardless of the used classifier. For low-performing holdouts, the attributes are more equally sampled, which introduces a bias and decreases the classification accuracy.

This feature could be formally analysed by calculating the Fisher’s ratio and the fold change of the attributes to construct the discriminatory plot, as shown in Fig. (**9**). The Fisher’s ratio (Eq. 8) is defined as the ratio of the variance between classes to the variance within classes:


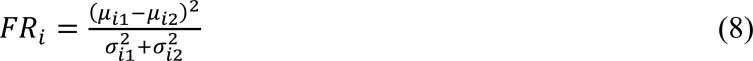


In other words, Fisher’s ratio detects the attributes that best separate the classes and are homogeneous within classes (with low intra-class variance). On the other hand, the fold change measures the inter-class distance between the distribution centers in both classes (Eq. 9):


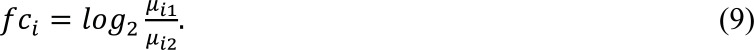


Fig. (**8**) shows that the sequence profile has very high variability in the Fisher’s ratio but almost no variability in the fold change. This implies a very high intra-class variation, while the mutation attributes have very high inter-class variations. Because of that, the combination of mutation attributes and attributes of the sequence offers high discriminatory power. It also explains why the high-performing holdouts contain these attributes, as shown in Fig. (**8**). Further, in the ranking of discriminatory power are evolutionary information and structural information attributes, both providing strong inter-class discrimination.

Analysing the discriminative power of various attributes provides deeper insights into the performance of other popular bioinformatics tools published in the literature compared to Holdout nsSNV. Table **3** summarizes the results of this comparison with other publicly available algorithms, such as PROVEAN, Mutation Taster, CADD, PPH-2, SNP & GO, PhD-SNP, PredictSNP, MAPP, Meta-SNP and CONDEL. Our holdout SAV algorithm ranks first among the methods listed in Table **3** and is mainly based on a similar philosophy.

The holdout nsSNV method is followed in terms of performance by the tools PhD-SNP, Meta-SNP and SNP & GO. The PhD-SNP and SNP & GO algorithms use a combination of sequence and mutation information to train the classifiers. Based on the distinctiveness analysis, both algorithms use the variables with the greatest predictive capacity. In our opinion, the Holdout nsSNV algorithm outperforms PhD-SNP and its performance due to the use of consensus, which reduces bias and increases precision. On the other hand, despite the use of mutation and sequence information, the SNP & GO algorithm also includes other features, such as evolutionary, stability or structural information, which introduce some bias due to noise or uncertainty. Therefore, the performance of this method is slightly lower than that presented here. The fact that Meta-SNP uses consensus could explain its better performance. It would be interesting to further investigate meta-SNP, including Fisher index analysis, to improve prediction accuracy.

PROVEAN, CADD MAPP, and PolyPhen-2 use the variables with the greatest discriminatory power. However, its accuracy is lower than ours due to the use of classifiers that are relatively sensitive to noise. Finally, it is worth mentioning the tools PredictSNP and CONDEL, which, despite using consensus, have no way to distinguish between high and low prediction variables. Therefore, the overall accuracy of this method is reduced due to the introduction of noise into the classification. An interesting feature, further supported by the analysis of the training performance of the Holdout Sampler, is the fact that the least squares classification methods, such as ELM or those included in CADD, PROVEAN, PPH-2, show lower accuracy compared to other RF-like methods. The comparison of the performance of different methods is based on the results published in the corresponding references. Ideally, a comparison would be made using the same data set applied to each method. In practice, this is very difficult for various reasons and is very rarely done in the literature (unavailability of the codes, unavailability of the data used for training with other methods to eliminate bias, *etc*.). Perfect blind testing must be performed on a completely new data set that has not been used by existing machine learning-based methods in the past. The best option would be to apply our method in the context of the Critical Assessment of Genome Interpretation (CAGI), a community experiment to objectively evaluate computational methods for predicting the phenotypic effects of genomic variation. CAGI participants receive experimentally studied genetic variants (not yet published) and make blind predictions about the resulting phenotype. We plan to participate in the CAGI experiment to blindly test the performance of our method and compare it with other leading methods. This is a perfect way to blind-test our methodology. The field of computational genomics is rapidly developing, with a variety of new databases, tools, and novel machine learning-based approaches being published regularly. Since it is difficult to cite all recently published papers, we refer the reader to several review papers, including papers that review applications of AI deep learning-based methods in this field [63-67].

It is worth also mentioning the Genomic Evolutionary Rate Profiling (GERP) [68] strategy for creating position-specific metrics of evolutionary constraints and the VarSome tool for the analysis of human genetic variations. These methods are very important in studying human genetic variants.

## 4 DISCUSSION

This paper presents a method that can predict the deleterious effects of certain types of SAVs by utilizing an advanced deep sampling procedure. Due to the high degree of uncertainty, this method is important for reducing bias and improving the accuracy of predictions. The strategy comprises of examining a set of classifier structures and boundary conditions for diverse holdouts (data bags) during the training phase. The finest performing holdouts are chosen to develop a consensus-based classifier. Our prediction system has been trained and blindly validated utilizing a k-fold (1 ≤ k ≤5) cross-validation method. Our methodology beats other consensus-based methods and gives strong, highly accurate outcomes with small standard deviations among folds. The predominance of our approach is based on the utilization of a tree of holdouts, where distinctive machine learning methods are inspected with diverse boundary conditions or distinctive predictive characteristics. This pre-parametrization permits us to construct a consensus classifier based on the leading holdouts. Nevertheless, it is important to keep in mind the inherent limitations of the proposed methodology that is based on AI algorithms, that is, no physical model exists to predict the effect of a mutation. In this way, its performance will be clearly impacted by the database that we have at our disposal. The main problems encountered in using machine learning models include the sparsity of the data, *i.e*., a situation where a large amount of data is missing or incomplete, resulting in gaps in a dataset, and imbalanced data, *i.e.,* with skewed class proportions between elements in the dataset. These are good news since machine learning methods typically improve when the data available for sampling increases, and rapid growth of genomic data significantly improves the reliability of AI big data-based methods.

## CONCLUSION

Our Consensus Holdout Sampler outflanks other popular algorithms, and gives excellent results, highly accurate with low standard deviation. The advantage of our method emerges from using a tree of holdouts, where diverse LM/AI-based programs are sampled in diverse ways.

## Figures and Tables

**Fig. (1):**
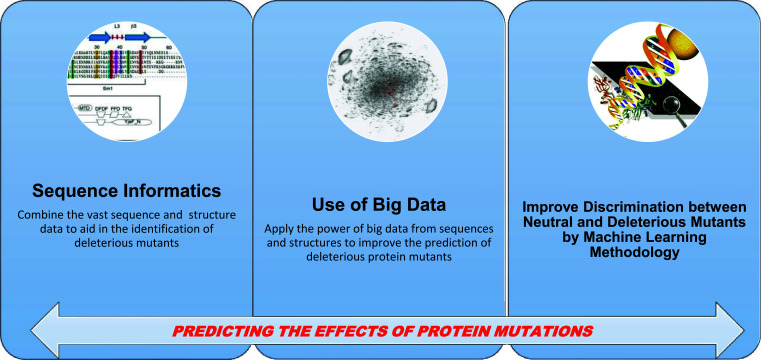
General flowchart of the paper.

**Fig. (2):**
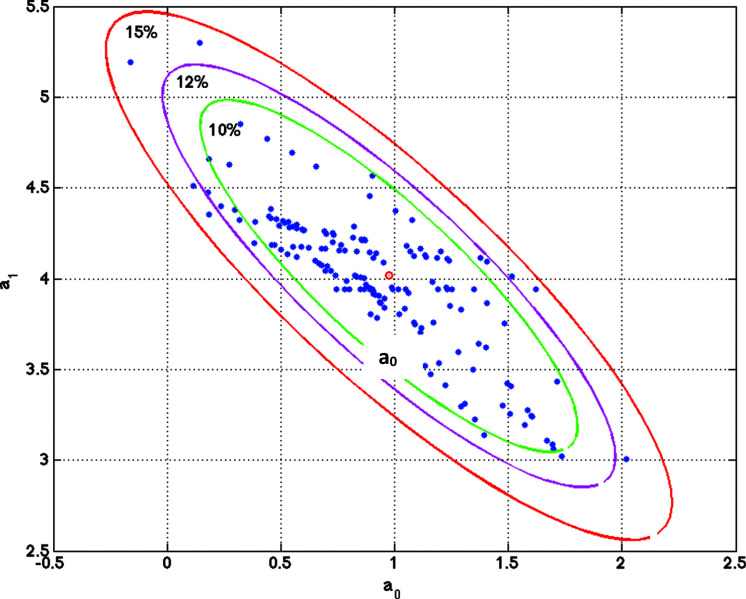
Illustration of a linear regression model. Ellipses of uncertainty for different relative misfits ranging from 10% to 15% and different sets of models are shown. This simple case is very important to understand that the solution to the problem of prediction of nsSNV effects is not unique. There are sets of different nsSNV attributes that could predict the observed effect within a given tolerance. In our case, we introduce the Holdout Sampler as a robust way of sampling the highly discriminatory attributes that best predict the observed effects of nsSNV.

**Fig. (3):**
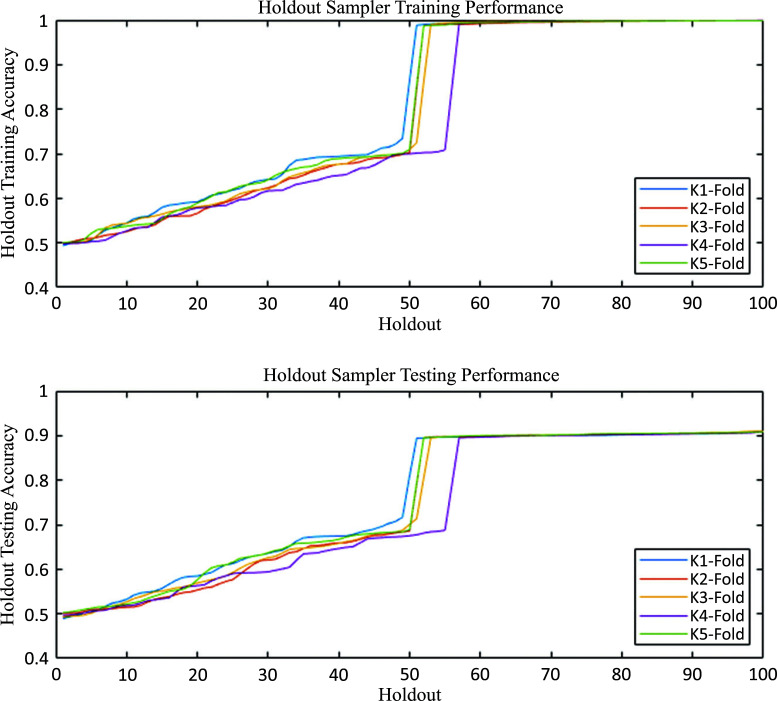
Holdout sampler training and testing accuracy distribution functions. It is possible to visualize two clear performance behaviours depending on the choice of the classifier (ELM or RF) and protein attributes that are selected to represent single amino acid polymorphisms. It is important to note that, in this case, the classifier in each data bag has been chosen randomly among these two classifiers. Besides, although not shown, the holdout RF approach provides better results than the simple RF.

**Fig. (4):**
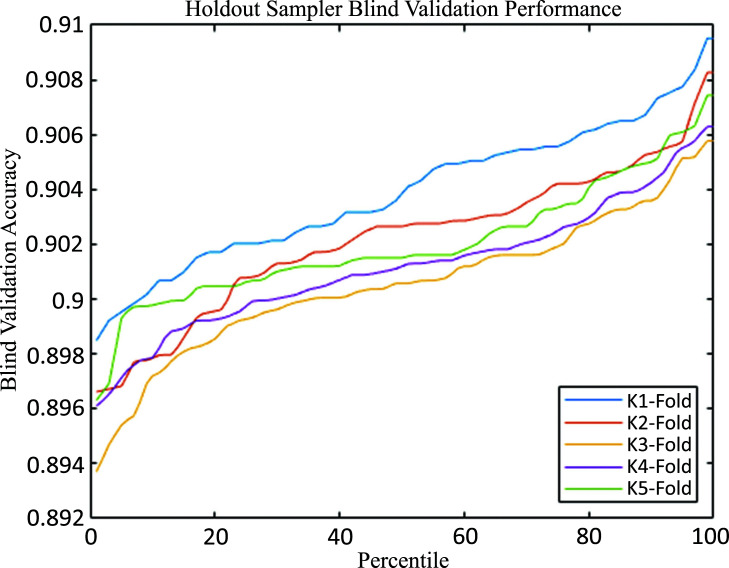
Cumulative distribution function of accuracies for the blind validation for the holdout sampler. The robustness of the algorithm and the lack of bias are manifested by the fact that the CDFs are smooth over a small accuracy range for different folds. The median accuracy in the different folds is given by the percentile 50, which is always higher than 0.9.

**Fig. (7):**
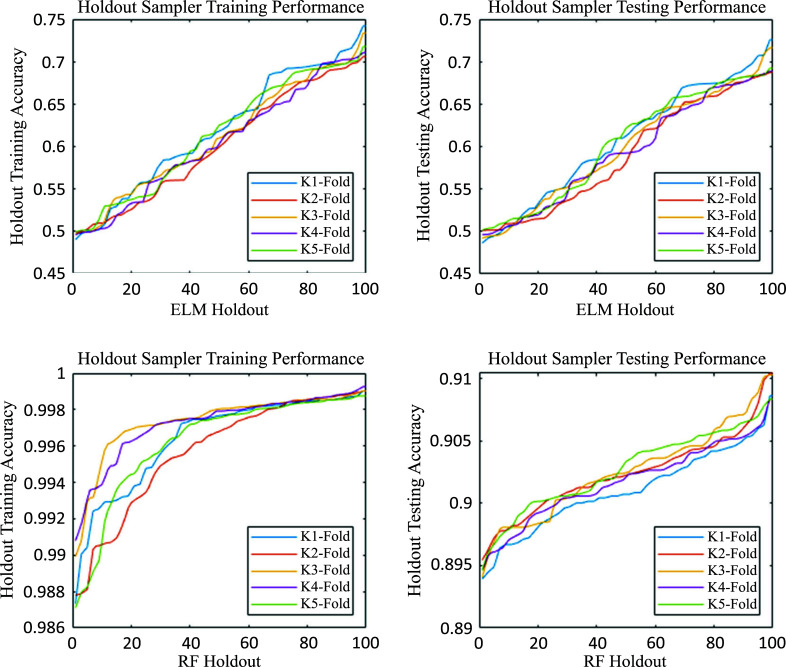
Performance of ELM and RF holdouts at the training and testing stages. It is possible to observe the remarkable difference in the performance of both classifiers, where the inherent robustness of RF significantly outperforms ELM. The testing median accuracy of ELM is around 0.6, while for RF, it is greater than 0.9. Therefore, the holdout RF algorithm clearly outperforms the ELM holdouts.

**Fig. (8):**
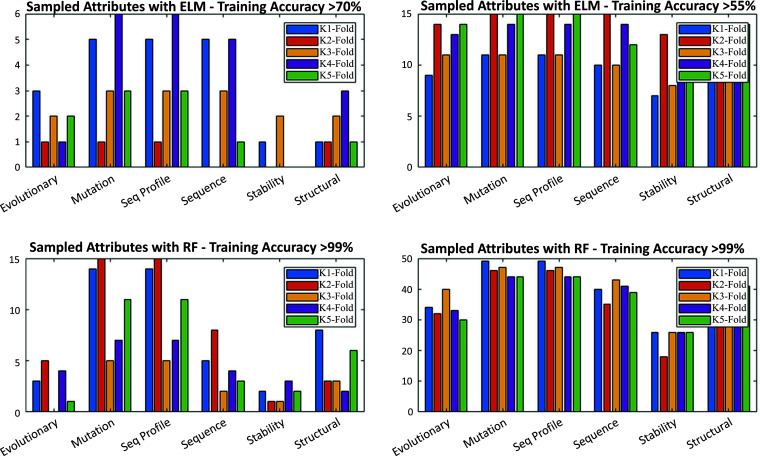
Frequency (posterior analysis) of the high discriminatory attributes (Evolutionary, mutation, sequence profile, sequence, stability, structural attributes) identified in the sampling for the ELM classifiers (upper figures) or RF classifiers (upper figures). The figures on the right correspond to a training stage of a deep learning algorithm without fixing the classifier’s architecture and on the left to a random selection of the attributes utilized to infer the class.

**Fig. (9):**
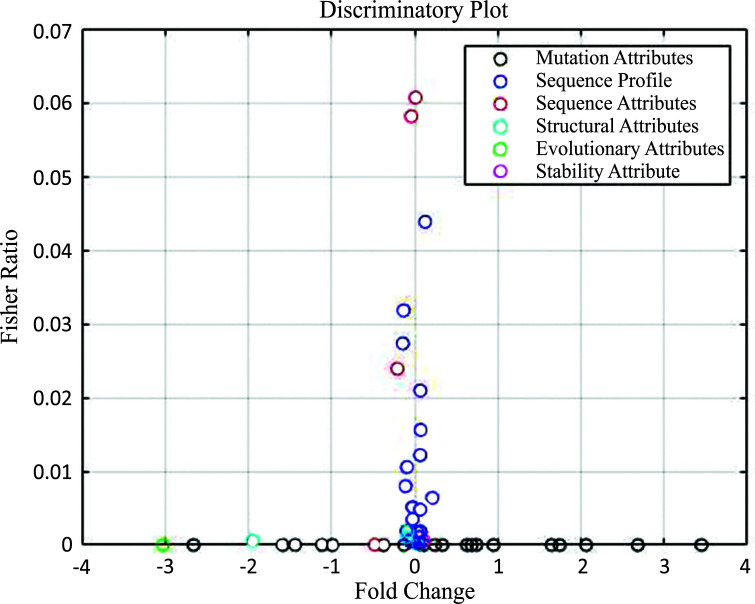
Discriminatory plot of the attributes utilized as boundary decision attributes in the holdout sampler. The combination of mutation information and sequence attributes and profile offers the most discriminatory power in the training and blind validation as measured by Fisher’s ratio.

**Table 1:** Training performance of holdout- nsSNV prediction tool in each individual K-Fold.

**K-Fold**	**Mean Accuracy**	**Median Accuracy**	**Accuracy Std**	**Accuracy ** **Uncertainty**	**Minimum ** **Accuracy**	**Maximum ** **Accuracy**
1	**75.40**	**80.99**	15.56	28.82	48.63	**90.86**
2	**74.31**	**68.58**	16.36	32.55	50.11	**91.04**
3	**74.46**	**70.08**	15.98	30.98	49.22	**91.03**
4	**72.93**	**67.52**	16.08	31.11	49.63	**90.83**
5	**74.97**	**68.83**	15.78	28.72	50.16	**90.83**
Overall	**74.41**	**71.20**	15.95	30.44	49.55	**90.92**

**Table 2:** Blind validation performance of the holdout- nsSNV for each individual K-Fold.

**K-Fold**	**Mean Accuracy**	**Median Accuracy**	**Accuracy Std**	**Accuracy ** **Uncertainty**	**Minimum ** **Accuracy**	**Maximum ** **Accuracy**
1	**90.38**	**90.38**	0.26	0.35	89.85	**90.95**
2	**90.21**	**90.27**	0.27	0.34	89.66	**90.83**
3	**90.05**	**90.06**	0.26	0.27	89.4	**90.58**
4	**90.12**	**90.12**	0.23	0.28	89.61	**90.63**
5	**90.20**	**90.15**	0.22	0.27	89.63	**90.74**
Overall	**90.19**	**90.20**	0.25	0.30	89.63	**90.75**

**Table 3:** Comparison of performance of holdout- nsSNV sampler with other most popular prediction tools.

**Performance Metrics**	**PROVEAN**	**CADD**	**PPH-2**	**SNP & GO**	**PhD-SNP**	**PredictSNP**	**MAPP**	**Meta-SNP**	**CONDEL**	**Holdout-SAV**
Dataset	SwissProt	UniProt HumVar	SwissProt	SwissProt	SwissProt	SwissProt	SwissProt	SwissProt	SwissProt	SwissProt
Accuracy	79%	76%	70%	83%	88%	75%	71%	**87%**	75%	**90%**
MC	0.74	*Not reported	0.41	0.67	0.72	0.49	0.41	0.74	0.51	**0.80**
AUC	0.85	0.86	0.78	**0.91**	**0.91**	0.81	0.77	0.91	0.82	0.90
Refs.	[13]	[20]	[21]	[22]	[23]	[24]	[25]	[35]	[36]	-

## Data Availability

The MATLAB program used to get our results can be obtained by contacting the authors. We are working on the development of the publicly available server that will predict the effects of amino acid mutations.

## LIST OF ABBREVIATIONS

AUCs: Areas Under the Curve
BLASTP: Basic Local Alignment Search Tool
CAGI: Critical Assessment of Genome Interpretation
ELM: Extreme Learning Machine
GERP: Genomic Evolutionary Rate Profiling
MCC: Mathews Correlation Coefficient
nsSNVs: nonsynonymous Single Nucleotide Variants
PANTHER: Proteins Annotation through Evolutionary Relationships
RF: Random Forest
ROC: Receiver Operating Characteristic
SAPs: Single Amino Acid Polymorphisms
SNV: Single Nucleotide Variations
SVM: Support Vector Machines
